# Transneuronal Degeneration in the Visual Pathway of Rats following Acute Retinal Ischemia/Reperfusion

**DOI:** 10.1155/2021/2629150

**Published:** 2021-12-07

**Authors:** Yanyan Fu, Tu Hu, Qianyue Zhang, Shuhan Meng, Ying Lu, Aiqun Xiang, Yewei Yin, Yuanjun Li, Jiayu Song, Dan Wen

**Affiliations:** ^1^Department of Ophthalmology, Xiangya Hospital, Central South University, Changsha, 410008 Hunan, China; ^2^Hunan Key Laboratory of Ophthalmology, China; ^3^Central South University National Clinical Research Center for Geriatric Disorders Xiangya Hospital, China

## Abstract

The maintenance of visual function not only requires the normal structure and function of neurons but also depends on the effective signal propagation of synapses in visual pathways. Synapses emerge alterations of plasticity in the early stages of neuronal damage and affect signal transmission, which leads to transneuronal degeneration. In the present study, rat model of acute retinal ischemia/reperfusion (RI/R) was established to observe the morphological changes of neuronal soma and synapses in the inner plexiform layer (IPL), outer plexiform layer (OPL), and dorsal lateral geniculate nucleus (dLGN) after retinal injury. We found transneuronal degeneration in the visual pathways following RI/R concretely presented as edema and mitochondrial hyperplasia of neuronal soma in retina, demyelination, and heterotypic protein clusters of axons in LGN. Meanwhile, small immature synapses formed, and there are asynchronous changes between pre- and postsynaptic components in synapses. This evidence demonstrated that transneuronal degeneration exists in RI/R injury, which may be one of the key reasons for the progressive deterioration of visual function after the injury is removed.

## 1. Introduction

Retinal ischemia/reperfusion (RI/R) causes traumatic tissue and progressive degeneration of retina ganglion cells (RGCs) [1]. These pathological events have been considered as the major cause of RI/R-induced visual dysfunction for decades [2]. Intervention measures that only compete against the mechanisms of in situ RGC death cannot recover the degenerated visual function following RI/R [3, 4]. Thus, there may be some other key factors that contribute to visual dysfunction following RI/R injury.

Neuroscience studies have demonstrated the significant transneuronal degeneration in neurodegenerative diseases [5, 6], which can be manifested as cellular edema, apoptosis, and axonal degeneration [7, 8]. These remote pathological events were usually neglected but cause functional damage that ultimately affects prognosis. Diffusion tensor imaging (DTI) reveals that the white matter of the visual pathways demonstrates damage proportional to the amount of structural and functional changes in the optic nerve [9]. Functional magnetic resonance imaging (fMRI) further described the blood-flow abnormalities in the brain of patients with primary open-angle glaucoma [10]. Pathological studies have shown that the atrophy of the LGN plate, the deformation of neuronal dendrites, decreases of metabolic activity in LGN neurons, and even the thickness of the primary visual cortex emerged a significant reduction [11, 12]. A molecular study showed that the NLRP3 inflammasome can be activated by ATP-P2X7-NLRP3 signal channel after optic nerve damage and involved in the secondary degeneration of neurons in the V1 region of the visual cortex [13]. These studies suggested that the primary retinal neuronal damage has profound effects on synaptic-linked distant neurons. However, what detailed mechanisms might involve in these transneuronal degenerations?

Scholars have found the synaptic plasticity alterations at the early stage of neurological diseases such as Alzheimer's disease (AD), epilepsy, and convulsions [6, 8, 14], which probably contribute to the transneuronal degeneration and be responsible for transferring damage signal between neurons [15, 16]. For further exploration, Kim et al. [17] revealed that tau protein deposition within axons and spread along synapse in corticopontine pathways can promote transneuronal degeneration in the frontotemporal lobar. Thus, a hypothesis is raised that the presynaptic terminal changes may break down the routing and sorting mechanisms for the cytoskeletal protein tau, which promote the formation of initial neurofibrillary pathology in the postsynaptic neurons via anterograde transneuronal mechanisms. In Wolfram syndrome, the downregulation of kinesin engine protein in synaptic terminals slows down the speed of axoplasmic transport, which leads to the decreasing in presynaptic vesicles and SYN, and this event participated in the extensive neuronal loss in LGN [18]. In addition, synapses can transfer damage signals by regulating the levels of cytokines such as inflammatory factors [19], brain-derived neurotrophic factor (BDNF) [20], and vascular endothelial growth factor (VEGF) [21]. Furthermore, synapses can also affect distal neurons by remodeling the dendritic structures and increasing the axon bundles [22]. Moreover, the alteration of mitochondria proliferation in axon terminus has profound effects on distant neurons [23]. Recently, other researchers found that SYN increased following high intraocular pressure- (HIOP-) induced retinal injuries [22, 24]. Previous studies revealed that SYN promoted the release of excitotoxic glutamate, which enhanced the activity of N-methyl-D-aspartate (NMDA). This event led to the accumulation of soluble amyloid-*β* (A*β*) in hippocampal excitatory neurons [25]. These studies demonstrated that transneuronal degeneration has a strong correlation with retinal synaptic plasticity.

Until now, the details of transneuronal degeneration after RI/R have not been elucidated yet. In this study, we established the acute RI/R model of rats and summarized the temporal and spatial alterations of neurons and synapses in OPL, IPL, and LGN after RI/R injury. This study may provide more detailed pathology data in the visual pathway and more reliable intervention time and targets.

## 2. Materials and Methods

### 2.1. Animals and RI/R Model

One hundred and twenty adult Sprague-Dawley (SD) rats (female: 250-300 g, 9-10 weeks old) were purchased from the animal center of Central South University (License number: SCUD (Changsha) 2011-0004, Changsha, China). All animals were housed in clean level conditions with free access to food and water under the temperature of 22 ± 1°C, the humidity of 55 ± 5%, and a 12-hour light/dark cycle. All experimental procedures were reviewed and approved by the Animal Care and Use Committees of the Laboratory Animal Research Center at Xiangya Medical School of Central South University.

All animals were randomly and equally divided into five experimental procedures (for retrograde tracing, HE staining, immunohistochemistry, western blot analysis, and transmission electron microscope), and twenty-four animals were included in each procedure. We have eight groups in the first four experimental procedures, which means rats allowed to survive, respectively, at 2 hours, 6 hours, 12 hours, 1 day, 3 days, 7 days, and 14 days after injury (plus the untreated control groups, a total of eight observation groups). For transmission electron microscope (TEM), we have four experimental observation times, respectively, at controls, 12 hours, 3 days, 7 days, and 14 days after injury, and three animals were used for each observation time (*n* = 3 rats, 6 eyes).

All animals in the RI/R group were treated following the procedures previously described [26]; the rats were anesthetized with a 2% pentobarbital sodium (0.3 mL/100 g). A 30-gauge intravenous infusion needle connected to the installation instrument with normal saline was inserted into the anterior chambers of the eyes. The intraocular pressure (IOP) was slowly elevated to 14.63 kPa (110 mmHg), maintained for 60 min, and then gradually lowered to normal pressure.

### 2.2. Tissue Preparation

The retinal flats were used for retrograde tracing. First, the eyecups were prepared following perfusion. Next, the eyecups were fixed in 4% paraformaldehyde for 1 h at room temperature, and the retina was detached from the eyecups. To stretch the retinas flat, they were placed with ganglion cell layers facing upward on microscope slides and incised with four 3 mm long cuts at their superior, inferior, temporal, and nasal sides. Finally, the retinas were cleaned using a soft brush, covered with antifade aqueous mounting medium, and covers lipped.

The frozen sections were used for HE staining and immunohistochemistry. The eyecups or brain tissue were postfixed, immersed in ascending sucrose. After the dehydration was completed, the tissues were buried in the O.C.T. compound (SAKURA, USA), and stored at -20°C, and prepared for frozen sections. The eyecups were subjected to sagittal sections with 5 *μ*m thickness, and select sections with the optic nerve for standby. And brain tissue was subjected to coronary sections with 5 *μ*m thickness, and select sections with the dLGN for standby.

For western blotting, retinas and dLGN were dissected from deeply anesthetized rats and then weighed and quickly frozen on dry ice and stored at 80C for further homogenization.

For transmission electron microscopy (TEM), retinas and dLGN were dissected from perfused rats and rinsed with clean saline and then fixed in 2.5% pentanediol solution (tissue fixation solution for TEM) for electroscopic tissue sections.

### 2.3. Counting of RGCs

For retrograde labeling of RGCs, 4 days before sacrifice, Cholera Toxin B subunit-FITC (CTB-FITC, 3 *μ*L of 5% solution, Cat# abs80001, Absin, China) was introduced bilaterally into the dLGN according to the dLGN stereotaxic coordinates (bregma: 4.25 ± 0.18 mm, lateral: 3.00 ± 0.01 mm, and depth: 1.81 ± 0.13 mm; Song et al., 2019) using the stereotaxic instrument (RWD Life Science Co., Ltd., Shenzhen, Guangdong Province, China). After the perfusion, the eyeballs were dissected from deeply anesthetized rats, and the anterior segments were removed, and the posterior segments were fixed in 4% paraformaldehyde (0.1 M PB, pH 7.4) for 30 min. The retina was then isolated, and the retinal flat was as described above. RGCs were counted as previously reported [27]. Briefly, five fields for each quadrant (twenty in total) were taken within the central periphery of the retina regions (2 mm from the optic disc, Supplementary Figure 1) in each retina flat. CTB-labeled RGCs were counted at 400x magnification in each field; the mean of the 20 visual fields represents the number of this one retina. Corresponding regions from each retina of experimental and control groups were used for counting.

### 2.4. Hematoxylin and Eosin Staining

HE staining was conducted according to routine protocols [17]. The 5 *μ*m eyecup sections with the optic nerve were rewarmed and fixed in 4% paraformaldehyde for 15 min and then rinsed in double-distilled water (DDW). Then, the sections were stained with hematoxylin solution for 10 min followed by 1% acid ethanol (1% HCl in 70% ethanol) for 30 s and then rinsed in DDW. Then, the eyecup sections were stained with eosin solution for 1 min and followed by dehydration with graded alcohol (80%, 95%, and 100%) and clearing in xylene. The tissue surface was covered with glycerol, cover slides, and stored for morphological evaluation. Five sections were taken from each rat and observed under 200x microscopic magnification. The visual field of the central retina region (near the optic nerve) was selected, and the pathological image analysis software (CMIAS system) was used to measure the thickness of retinal OPL and IPL.

### 2.5. Immunohistochemistry

The brain tissue sections with the dLGN and the eyecup sections with the optic nerve were collected for immunofluorescent labeling. These sections were rewarmed and washed several times in PBS and then incubated with the anti-rabbit synaptophysin (1 : 500, Abcam, ab32127) and anti-mouse PSD-95 (1 : 500, Abcam, ab18258) overnight at 4°C. After several washes with PBS, sections were incubated with donkey anti-rabbit Alexa 594 (Jackson ImmunoResearch, 711-585-152) and goat anti-mouse Alexa 488 (Jackson ImmunoResearch, 115-545-062) for 2 hours at room temperature, followed by 0.5 *μ*g/mL DAPI (Thermo Fisher Scientific) in PBS for 5 minutes at room temperature before mounting. Five sections were taken from each rat and observed under 200x and 400x magnification immunofluorescence microscopy; the exposure time for eye sections was kept at 1 s and brain tissue sections at 2-3 s. Finally, use the Image J analysis software to measure the average grayscale values of positive products in retinal IPL and OPL.

### 2.6. Western Blotting

As previously detailed [28], tissue samples were homogenized by sonication on ice in RIPA buffer containing a cocktail of protease inhibitors (Sigma, MO, USA). Sonication-digested homogenates were treated with centrifugation, protein concentration determination, and degeneration, respectively. Total protein in tissue extracts was measured using a standard BCA assay (Pierce). Tissue extract proteins were resuspended in 5× sample buffer (60 mM Tris–HCl pH 7.4, 25% glycerol, 2% SDS, 14.4 mM 2-mercaptoethanol, and 0.1% bromophenol blue) at a 4 : 1 ratio, boiled for 5 min, and resolved by SDS-PAGE. Proteins were transferred onto a nitrocellulose membrane, and blots were stained with Ponceau S (Sigma, St. Louis, MO, USA) to visualize the protein bands and ensure equal protein loading and uniform transfer. Blots were washed and blocked for 45 min with 5% nondried skim milk in TBST buffer. Blots were then probed for 24 h using anti-rabbit synaptophysin (1 : 500, Abcam, ab32127) and anti-mouse PSD-95 (1 : 500, Abcam, ab18258) and Actin (Sigma, St. Louis, MO, USA). Blots were then probed with horseradish peroxidase- (HRP-) conjugated goat anti-rabbit secondary antibody and HRP-donkey anti-mouse secondary antibody. Bound antibodies were detected using an enhanced chemiluminescence system (Amersham) and X-ray film. Relative intensity was measured using an ImageMaster®VDS (Pharmacia Biotech), and the fold changes in these protein levels are indicated below the blot. Results are representative of five independent experiments. Data are expressed as mean ± SD.

### 2.7. Transmission Electron Microscopy

Electron microscopy was conducted using retinal and dLGN electron microscopic sections from controls and postsurgery (three rats per group). Retina and dLGN tissue were cut into 1 mm^3^ cube with a vibratome and rinsed with clean saline. Tissues were fixed in 2.5% glutarol solution for 1 hour at room temperature or 3 hours at 4°C and then with 1% osmium tetroxide in 0.1 mmol/L cacodylate buffer for 2 hours. After rinsing with DDW, sections were treated with 1% aqueous uranyl acetate overnight, dehydrated in ethanol solutions of increasing concentration, up to 100%, followed by dry acetone, and then embedded in durcupan ACM. Ultrathin sections (0.1 *μ*m) were cut and mounted on Formvar-coated slot grids, stained with 3% lead citrate, and examined with a Zeiss transmission EM (Zeiss).

### 2.8. Statistical Analysis

The IBM SPSS Statistics (23.0) software was applied in this study; all data are expressed as means ± SD. Comparisons between the control group, 2 hours, 6 hours,12 hours, 1 day, 3 days, 7 days, and 14 days were performed using the one-way ANOVA test, and Student's *t*-test was used in pairwise comparisons. Differences with *P* < 0.05 were considered statistically significant.

## 3. Results

### 3.1. Changes of Neuronal Soma in Visual Pathways following RI/R

#### 3.1.1. RGC

**Figure 1:**
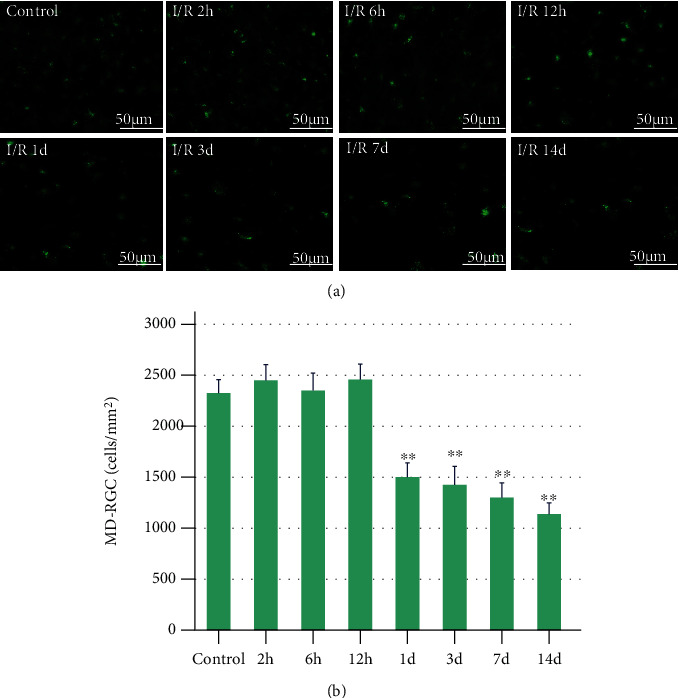
The mean density of RGC retrograde labeled by CTB (scale bar = 50 *μ*m). (a) I/R-2 h to 14 d: representative photomicrographs were taken at high magnification of rats euthanized 2 hours, 6 hours, 12 hours, 1 day, 3 days, 7 days, and 14 days post-RI/R injury. (b) MD-RGCs: I/R-1 d, I/R-3 d, I/R-7 d, and I/R-14 d (rats euthanized 1 day, 3 days, 7 days, and 14 days post-RI/R injury) were less than those in the control group (*P* < 0.001). I/R-2 h: rats euthanized 2 hours post-RI/R injury; I/R-6 h: rats euthanized 6 hours post-RI/R injury; I/R-12 h: rats euthanized 12 hours post-RI/R injury; I/R-1 d: rats euthanized 1 day post-RI/R injury; I/R-1 d: rats euthanized 3 days post-RI/R injury; I/R-7 d: rats euthanized 7 days post-RI/R injury; I/R-14 d: rats euthanized 14 days post-RI/R injury. ^∗^*P* < 0.05 indicates that there were statistical differences when compared with the control groups. ^∗∗^*P* < 0.01 indicates that there were significant statistical differences when compared with the control groups.

*(1) Mean Density of CTB-Labeled Retinal Ganglion Cells*. We used cholera toxin subunit (CTB) as a retrograde tracer to mark RGCs, and the results obtained that the mean density of RGCs (MD-RGCs) in the peripheral retina was (2325 ± 133)/mm^2^ in the control group. And there was no significant difference between I/R-2 h (*P* = 0.825), I/R-6 h (*P* = 1.581), I/R-12 h (*P* = 1.304) groups, and control group in mean density. Specific values for the different groups are shown in Table 1. The mean density of RGCs in I/R-1 d, I/R-3 d, I/R-7 d, and I/R-14 d were less than those in the control group (*P* < 0.001) (Figures 1(a) and 1(b)).

**Figure 2:**
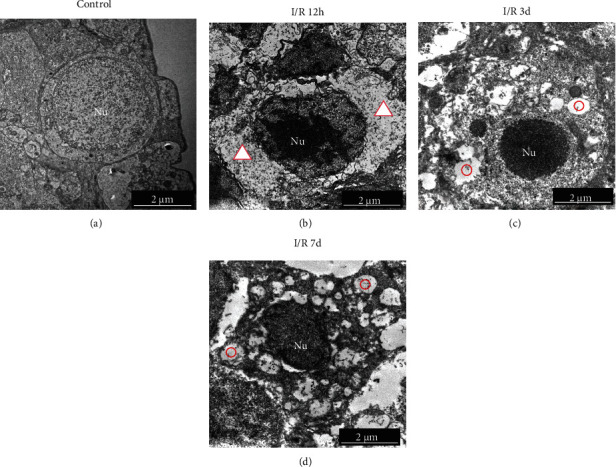
Changes of RGC ultrastructure after RI/R injury (10000×, bar = 2 *μ*m). (a) Control group, Nu: nucleus of RGCs: the nucleus of RGCs presented round shape with smooth nuclear contours and accounted for most volume in the normal physiological state. (b) I/R-12 h group, the morphology of RGCs at 12 hours post-RI/R injury: the cytoplasmic edema was visible (red triangle) and nuclear chromatin became condensed, but the aggregation of chromatin and fragmentation of nuclei did not occur (Nu). (c) I/R-3 d group, the morphology of RGCs at 3 days post-RI/R injury: the cytoplasmic vacuole formed in RGCs (red circle). The chromatin continues to aggregate, and the volume of the nucleus shrunk (Nu). (d) I/R-7 d group, the morphology of RGCs at 7 days post-RI/R injury: more vacuolar can be seen in the cytoplasm of RGCs (red circle), and the fragmentation of nuclei began to appear; both the chromatin and the organelles began to dissolve; these phenomena suggest necrosis in RGCs. I/R-12 h: rats euthanized 12 hours post-RI/R injury; I/R-3 d: rats euthanized 3 days post-RI/R injury; I/R-7 d: rats euthanized 7 days post-RI/R injury.

*(2) Changes of RGCs Ultrastructure following RI/R*. Under the TEM, normal RGCs showed round nuclei with smooth nuclear contours and account for a large proportion of volume in cell body (Figure 2(a)). At 12 hours after RI/R injury, we can see the cytoplasmic edema was prominent in RGCs (Figure 2(b), red triangle). Moreover, nuclear chromatin became condensed, but the aggregation of chromatin and fragmentation of nuclei did not occur (Figure 2(b), Nu). At 3 days after RI/R injury, the cytoplasmic vacuole (Figure 2(c), red circle) formed in RGCs. The chromatin continues to aggregate, and the volume of the nucleus shrunk (Figure 2(c), Nu). At 7 days after RI/R injury, more vacuole can be seen in the cytoplasm of RGCs, and the fragmentation of nuclei began to appear; both the chromatin and the organelles began to dissolve; these phenomena suggest necrosis in RGCs (Figure 2(d)).

#### 3.1.2. Bipolar Cells

**Figure 3:**
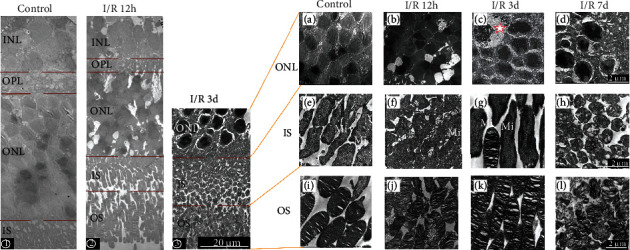
Ultrastructure changes of neurons in the retina after RI/R injury. ①–③: ultrastructure changes in each layer of the retina under 1000× TEM (bar = 20 *μ*m). ① Control group. The boundaries between different layers were clear. The nucleus in ONL and INL was neatly arranged with a polygonal cross-section. ② Ultrastructure of each layer in the retina at 12 hours post-RI/R injury. Layer demarcation becomes unclear, and the arrangement of cells becomes loose, and obvious macular edema was observed. ③ Ultrastructure of each layer in the retina at 3 days post-RI/R injury. The macular edema showed a slight trace of recovery, but cell morphology becomes irregular, and nuclear chromatin became condensed. (a–l) Ultrastructure changes in each layer of the retina under 10000× TEM (bar = 2 *μ*m). I/R-12 h: rats euthanized 12 hours post-RI/R injury; I/R-3 d: rats euthanized 3 days post-RI/R injury; I/R-7 d: rats euthanized 7 days post-RI/R injury. (a) Nucleus in ONL was neatly arranged with a polygonal cross-section. (e) Outside the ONL are the inner segments (ISs) of photoreceptor (PR) cells, and they were narrow and long, with a long spindle shape, were arranged orderly, and rich in mitochondria at the peripheral portion of the soma. (l) Outer segment (OS) of photoreceptor (PR) cells contained many flattened membranous structures called discs. In the normal state, the disc is neatly folded inside, with few gaps in between. (b, f, j) I/R-12 h group. (b) Some nucleus in ONL was replaced by vacuoles. (f) Edema in the IS made cells' volume increased significantly which decreased intercellular space. Mitochondria (Mi) were significantly increased and swelled. (j) OS swelled, the arrangement of membranous discs becomes disordered, and the space between membranous discs became widened. (c, g, k) I/R-3 d group. (c) Gliocyte proliferation in ONL (red star), cell edema, and chromatin aggregation allowed the nucleus to be deeply stained. (g) The edema in the IS showed a slight trace of recovery compared to the I/R-12 h group, while mitochondria continue to proliferate and hypertrophy (Mi). (k) The edema in the OS showed a slight trace of recovery. (d, h, l) I/R-7 d group. (d) Nucleus in ONL was lysed, and the number reduced. (h) Organelle was lysed, and the membrane was discontinuous in IS. (o) The membrane disc was loose, dissolved, and shrunk.

The nucleus of bipolar cells in INL was neatly arranged with a polygonal cross-section (Figure 3, ①). At 12 hours after RI/R injury, severe cellular edema of bipolar cells in the INL was observed, and some nuclei in INL were replaced by vacuoles (Figure 3, ②). At 3 days after injury, the macular edema of bipolar cells showed a slight trace of recovery but cell morphology becomes irregular and nuclear chromatin became condensed (Figure 3, ③). At 7 days after injury, the shape of bipolar cells became irregular, and the chromatin and the organelles began to dissolve, suggested the apoptosis of bipolar cells late after injury.

#### 3.1.3. Photoreceptor Cells

Nucleus of photoreceptor (PR) cells in ONL was neatly arranged with a polygonal cross-section (Figure 3(a)). Outside the ONL are the inner segments (ISs) of photoreceptor (PR) cells; they were narrow and long, with a long spindle shape, were arranged orderly, and rich in mitochondria at the peripheral portion of neuronal soma (Figure 3(e)). The outer segment (OS) of photoreceptor (PR) cells contained many flattened membranous structures called discs. In the normal state, the disc is neatly folded inside with few gaps in between (Figure 3(i)). At 12 hours after RI/R injury, many PR cells' nuclei were replaced by vacuoles (Figure 3(b)), and edema in the IS made cells' volume increased significantly. Mitochondria (Mi) were significantly increased and swelled (Figure 3(f)). The OS swelled, and the arrangement of membranous discs becomes disordered; the space between membranous discs became widened (Figure 3(j)). At 3 days after RI/R injury, we found gliocyte proliferation chromatin aggregation in ONL (Figure 3(c), red star). At 3 days after injury, the edema in the IS showed a slight trace of recovery but while mitochondria continue to proliferate and hypertrophy (Figure 3(g), Mi). Then, nuclei in ONL were lysed, and the number reduced at 7 days after injury (Figure 3(d)). Organelle was lysed, the membrane was discontinuous in IS (Figure 3(h)), and the membrane disc was loose, dissolved, and shrunk (Figure 3(l)).

#### 3.1.4. Neurons in LGN

**Figure 4:**
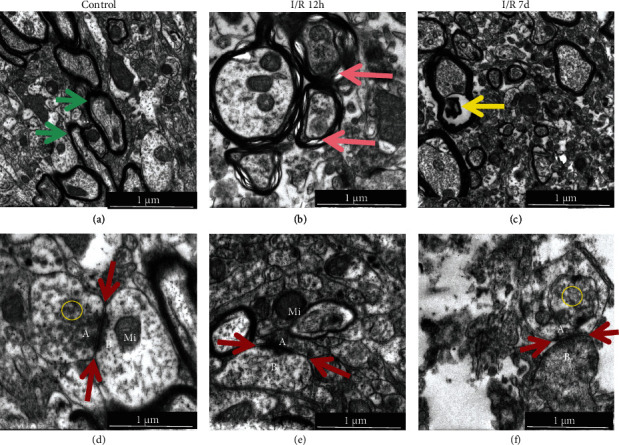
Ultrastructure changes of neurons in LGN after RI/R injury (TEM ×20000). I/R-12 h: rats euthanized 12 hours post-RI/R injury; I/R-3 d: rats euthanized 3 days post-RI/R injury; I/R-7 d: rats euthanized 7 days post-RI/R injury. (a) Control group: nerve fiber bundles in LGN were orderly arranged, axons were surrounded by a myelin sheath, and the endoneurium-encased myelin sheath and axons were clear and visible on the nerve fiber surface (green arrow). In normal conditions, axons were tightly surrounded and there is no space between axons and myelin. (b) I/R-12 h group: clear space (red arrow) appears in the myelin sheath suggesting early demyelination changes. The axons showed significant edema and mitochondrial hypertrophy and hyperplasia. (c) I/R-7 d group: there are heterotypic protein clusters (yellow arrow) within axons, and the diameter of axons decreased significantly; this is a phenomenon of axonal degeneration. (d) Control group, (red arrow): synaptic junction in LGN. The presynaptic (A) and postsynaptic (B) membranes are clear, presynaptic thickenings form a synaptic active zone (red arrow), and clusters of synaptic vesicles (yellow circle) and mitochondria were normal in size and structure (Mi). (e) I/R-12 h group: the synapsis in LGN showed significant edema which made the membrane of synapsis becomes thinner, and the synaptic active band becomes broadened (red arrow), whereas mitochondrial hyperplasia and hypertrophy in presynapses (Mi). (f) I/R-7 d group: membranes of the synapses were discontinuous, organelle was lysed, presynaptic vesicles reduced, and the synaptic structure became unclear.

Under normal physiological conditions, nerve fiber bundles in LGN were orderly arranged, axons were surrounded by a myelin sheath, and the endoneurium-encased myelin sheath and axons were clear and visible on the nerve fiber surface (green arrow). Axons were tightly surrounded, and there is no space between axons and myelin (Figure 4(a)). At 12 hours after RI/R injury, clear space (red arrow) appears in the myelin sheath representing early demyelination changes. The axons showed significant edema and mitochondrial hypertrophy and hyperplasia (Figure 4(b)). At 7 days after RI/R injury, there were heterotypic protein clusters (yellow arrow) within axons and the diameter of axons decreased significantly (Figure 4(c)).

### 3.2. Changes of Synapsis in Visual Pathways following RI/R Damage

#### 3.2.1. Changes in IPL and OPL following RI/R Damage

**Figure 5:**
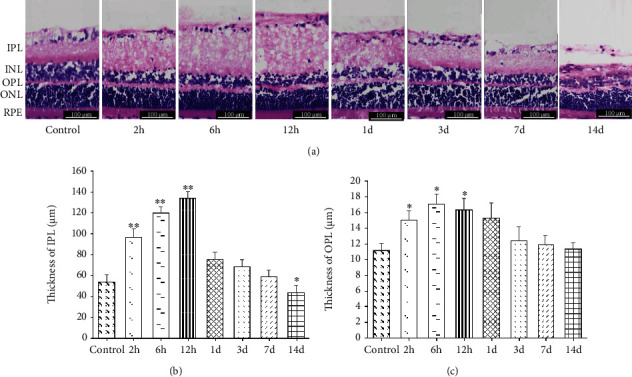
(a) Representative images of HE staining showed morphological alterations of neurons in the retina after RI/R injury (HE 200×, bar = 100 *μ*m). 2 h: rats euthanized 2 hours post-RI/R injury; 6 h: rats euthanized 6 hours post-RI/R injury; 12 h: rats euthanized 12 hours post-RI/R injury; 1 d: rats euthanized 1 day post-RI/R injury; 3 d: rats euthanized 3 days post-RI/R injury; 7 d: rats euthanized 7 days. CMIAS image analysis system was used to quantify the thickness of (b) IPL and (c) OPL, and optical microscopy images were captured using an inverted microscope at 200x magnification. IPL: inner plexiform layer; INL: inner nuclear layer; OPL: outer plexiform layer; ONL: outer nuclear layer; RPE: retinal pigment epithelium. ^∗^*P* < 0.05 indicates that there were statistical differences when compared with the control groups. ^∗∗^*P* < 0.01 indicates that there were significant statistical differences when compared with the control groups.

*(1) Morphological Changes in IPL and OPL*. Observation under the optical microscope revealed that a normal 10-week-old female rat's thickness was 54.03 ± 6.78 *μ*m for IPL and 11.16 ± 0.91 *μ*m for OPL (Figure 5(a), control). At 2 to 12 hours after RI/R injury, the thickness of IPL continues to increase (*P* < 0.01) and reach the peak of thickness at 12 hours (133.89 ± 6.65 *μ*m), which was 2.46 times of the control group. Subsequently, at 1 to 14 days after RI/R injury, the thickness of IPL continues to decrease, and it was significantly lower than the control group at 14 days (*P* = 0.032) (Figure 5(a)).

The same trend was observed in OPL, and at 2 to 12 hours after injury, the thickness of OPL was all greater than that in the control group (*P* < 0.05), 15.06 ± 1.17 *μ*m for the I/R-2 h group, 17.04 ± 1.32 *μ*m for the I/R-6 h group, and 16.35 ± 1.44 *μ*m for the I/R-12 h group. At 1 to 14 days after injury, thickness values of OPL showed a downward trend, 15.27 ± 1.93 *μ*m for the I/R-1 d group, 12.39 ± 1.77 *μ*m for the I/R-3 d group, 11.88 ± 1.21 *μ*m for the I/R-7 d group, and 11.34 ± 0.84 *μ*m for the I/R-14 d group (Figure 5(b)). The specific thickness values are shown in Table 2.

**Figure 6:**
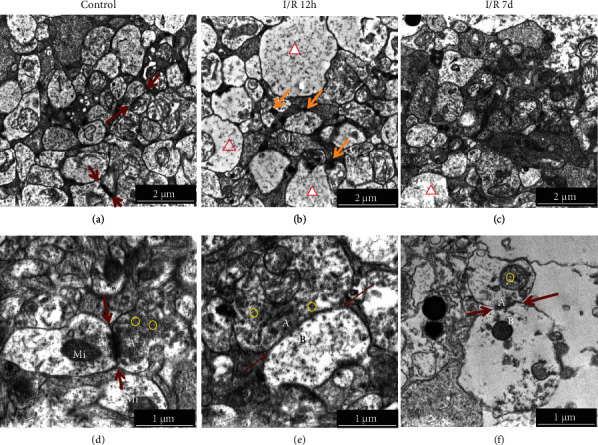
Ultrastructure changes in IPL and OPL after RI/R injury (TEM ×10000, ×20000). I/R-12 h: rats euthanized 12 hours post-RI/R injury; I/R-7 d: rats euthanized 7 days post-RI/R injury. (a) Control group (10000×; bar = 2 *μ*m): numerous synapses were evenly distributed (red arrow), and they are closely arranged and uniform in size and shape under normal circumstances. (d) Control group (20000×; bar = 1 *μ*m). The presynaptic (A) and postsynaptic (B) membranes are clear, presynaptic thickenings form a synaptic active zone (red arrow), and clusters of synaptic vesicles (yellow circle) and mitochondria were normal in size and structure (Mi). (b) I/R-12 h group (bar = 2 *μ*m): neurons in IPL and OPL showed significant edema (red triangle) and mitochondrial hyperplasia and hypertrophy (Mi). Many small immature synapses formed in the IPL and OPL (orange arrow). (e) I/R-12 h group (bar = 1 *μ*m): neuronal edema made the membrane of synapsis becomes thinner, presynaptic vesicles (yellow circle) increased in both IPL and OPL, and the synaptic active zone became wider and longer (red arrow). (c) I/R-7 d group (bar = 2 *μ*m): the small immature synapses disappeared, and neuronal edema gradually recovered, and aggregation of chromatin and fragmentation of nuclei can be observed. (f) I/R-7 d group (bar = 1 *μ*m): membranes of the synapses were discontinuous, cell was lysed, organelles were separated, presynaptic vesicles reduced (yellow circle), and the synaptic structure became unclear (red arrow). These phenomena suggest neuronal death in IPL and OPL.

Images of the TEM showed that numerous synapses were evenly distributed in the IPL and OPL, and they are closely arranged and uniform in size and shape under normal circumstances (Figure 6(a), red arrow). The presynaptic (Figure 6(d), A) and postsynaptic (Figure 6(d), B) membranes are clear, presynaptic thickenings form a synaptic active zone (Figure 6(d), red arrow), and clusters of synaptic vesicles (Figure 6(d), yellow circle) and mitochondria were normal in size and structure (Figure 6(d), Mi). At 12 hours after RI/R injury, neurons in IPL and OPL showed significant edema (Figure 6(b), red triangle), which made the membrane of synapsis becomes thinner, and mitochondrial hyperplasia and hypertrophy (Figure 6(b), Mi). Many small immature synapses were formed in the IPL and OPL (Figure 6(b), orange arrow). It can be seen under a higher magnification that neuronal edema and presynaptic vesicles (Figure 6(e), yellow circle) increased in both IPL and OPL, and the synaptic active zone became wider and longer (Figure 6(e), red arrow). At 7 days after RI/R injury, the small immature synapses disappeared and neuronal edema gradually recovered, and aggregation of chromatin and fragmentation of nuclei can be observed (Figure 6(c)). Under higher magnification, we found membranes of the synapses were discontinuous, cell was lysed, organelles were separated, presynaptic vesicles reduced (Figure 6(f), yellow circle), and the synaptic structure became unclear. These phenomena suggest neuronal death in IPL late after injury.

**Figure 7:**
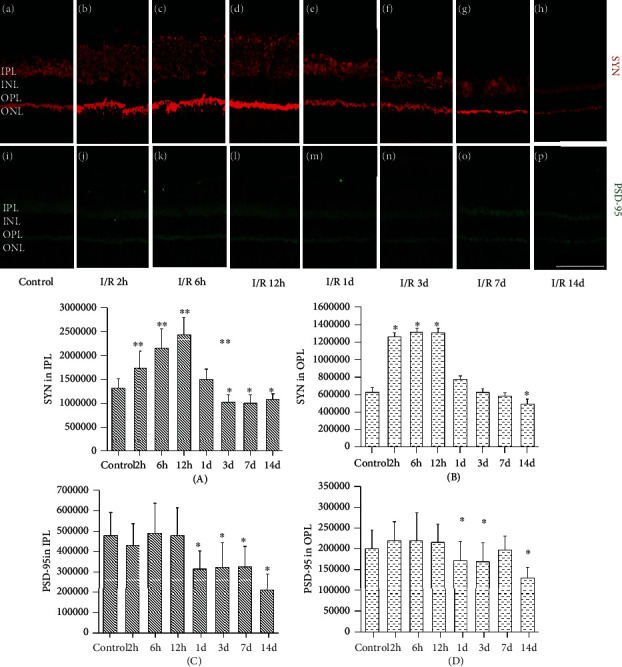
Immunofluorescence images of SYN and PSD-95 expression in the retina after RI/R injury (400x, bar = 50 *μ*m). I/R-2 h: rats euthanized 2 hours post-RI/R injury; I/R-6 h: rats euthanized 6 hours post-RI/R injury; I/R-12 h: rats euthanized 12 hours post-RI/R injury; I/R-1 d: rats euthanized 1 day post-RI/R injury; I/R-1 d: rats euthanized 3 days post-RI/R injury; I/R-7 d: rats euthanized 7 days post-RI/R injury; I/R-14 d: rats euthanized 14 days post-RI/R injury. SYN: synaptophysin (red); PSD-95: postsynaptic density-95 (green); IPL: inner plexiform layer; INL: inner nuclear layer; OPL: outer plexiform layer; ONL: outer nuclear layer. The Image J analysis software was used to determine the average gray value of the SYN expression in (A) IPL and (B) OPL, the average gray value of the PSD-95 expression in (C) IPL and (D) OPL. ^∗^*P* < 0.05 indicates that there were statistical differences when compared with the control groups. ^∗∗^*P* < 0.01 indicates that there were significant statistical differences when compared with the control groups.

*(2) SYN and PSD-95 Expression in IPL and OPL*. The expression of SYN and PSD-95 was mainly enriched in IPL and OPL; there are more positive products in IPL than in OPL (Figures 7(a)–7(p)). Quantitative analysis of gray value showed the expression of SYN in IPL increased significantly in I/R-2 h, I/R-6 h, and I/R-12 h groups (*P* = 0.012, 0.038, 0.022) (Figures 7(a)–7(d)). At 1 day after RI/R, the SYN expression began to decrease (Figure 7(e)) and keep this downtrend from 1 day to 14 days after the injury (Figures 7(e)–7(h)). The expression of SYN in the I/R-3 d, I/R-7 d, and I/R-14 d groups was all lower than the control group (*P* = 0.027, 0.008, 0.044) (Figure 7, A). The expression of PSD-95 was unchanged in I/R-2 h, I/R-6 h, and I/R-12 h groups when compared with the control group (*P* = 1.175, 1.838, 0.651) (Figures 7(i)–7(l), A) and began to decrease at 1 day after injury, whereas significantly lower than the control group in I/R-1 d, I/R-3 d, and I/R-14 d groups (*P* = 0.004, 0.018, 0.005) (Figures 7(m)–7(p), C).

Quantitative analysis of gray value showed the expression of SYN and PSD-95 in OPL had the same change trend as the expression in IPL. SYN increased significantly in I/R-2 h, I/R-6 h, and I/R-12 h groups (*P* = 0.012, 0.038, 0.022) (Figures 7(a)–7(d)) and began to decrease at 1 day after RI/R (Figure 7(e)). It kept this downtrend from 1 day to 14 days after the injury (Figures 7(e)–7(h)). The expression of SYN in the I/R-1 d, I/R-3 d, I/R-7 d, and I/R-14 d groups was all lower than the control group (*P* < 0.05) (Figure 7, C). Meanwhile, the expression of PSD-95 was unchanged in I/R-2 h, I/R-6 h, and I/R-12 h groups (*P* = 0.121, 0.852, 0.733) (Figures 7(i)–7(l), A) and began to decrease at 1 day after the injury, whereas significantly lower than the control group in I/R-1 d, I/R-3 d, and I/R-14 d groups (*P* = 0.011, 0.017, 0.042) (Figures 7(m)–7(p), C). It indicated that the expression of SYN in both IPL and OPL presents an asynchronous change with the expression of PSD-95.

**Figure 8:**
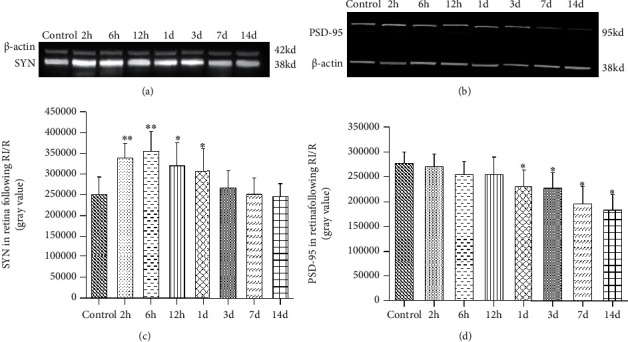
Western blotting results of SYN and PSD-95 expression in the retina after RI/R injury. (a) 2 h: rats euthanized 2 hours post-RI/R injury; 6 h: rats euthanized 6 hours post-RI/R injury; 12 h: rats euthanized 12 hours post-RI/R injury; 1 d: rats euthanized 1 day post-RI/R injury; 3 d: rats euthanized 3 days post-RI/R injury; 7 d: rats euthanized 7 days. Synaptophysin (SYN), a presynaptic vesicle protein, was significantly increased in the retina at 2 hours to 1 day after RI/R compared to the control group. From 3 days to 14 days, the SYN expression showed a downward trend. (b) PSD-95, a postsynaptic vesicle protein, remains unchanged at 2 hours to 12 hours after RI/R compared to the control group. The PSD-95 expression was significantly reduced in the retina at 1 day to 14 days after RI/R. Use the Image J analysis software to determine the average gray value of the (c) SYN expression and the (d) PSD-95 expression in retina. ^∗^*P* < 0.05 indicates that there were statistical differences when compared with the control groups. ^∗∗^*P* < 0.01 indicates that there were significant statistical differences when compared with the control groups.

In addition, western blot (WB) analysis also showed the same expression trend in the whole retina. SYN with the molecular weight of 38 kd was detected to be increased early after injury. From 2 hours to 1 day after the injury, the expression of SYN was significantly higher than control group (*P* < 0.001; *P* < 0.001; *P* = 0.009; *P* = 0.011). Then, from 1 d after injury, the expression began to decrease (*P* < 0.05), and this downward trend continued from 1 d to 14 d (Figures 8(a) and 8(c)). And PSD-95 with the molecular weight of 95 kd, expression remained unchanged from 2 hours to 12 hours after injury (*P* > 0.05). In I/R-1 d, I/R-3 d, I/R-7 d, and I/R-14 d groups, PSD-95 expression was significantly lower than the control group (*P* = 0.017, 0.003, 0.034, 0.001) (Figures 8(b) and 8(d)).

#### 3.2.2. Changes in dLGN following RI/R

*(1) Morphological Changes in dLGN*. Within the neuropil of LGN, synapses are evenly distributed and have a clear structure. The presynaptic and postsynaptic membranes were clear (Figure 4(d)), presynaptic thickenings form a synaptic active zone (Figure 4(d), red arrow), and clusters of synaptic vesicles (Figure 4(d), yellow circle) and mitochondria were normal in size and structure (Figure 4(d), Mi). At 12 hours after RI/R injury, multiple small newborn synapses formed within the neuropil, native synapsis in LGN showed significant edema which made the presynaptic and postsynaptic membrane become thinner, and the synaptic active band was broadened (Figure 4(e), red arrow), whereas mitochondrial hyperplasia and hypertrophy in presynapses (Figure 4(e), Mi). At 7 days after RI/R injury, membranes of the synapses were discontinuous, organelle was lysed, presynaptic vesicles reduced, and the synaptic structure became unclear (Figure 4(f)).

**Figure 9:**
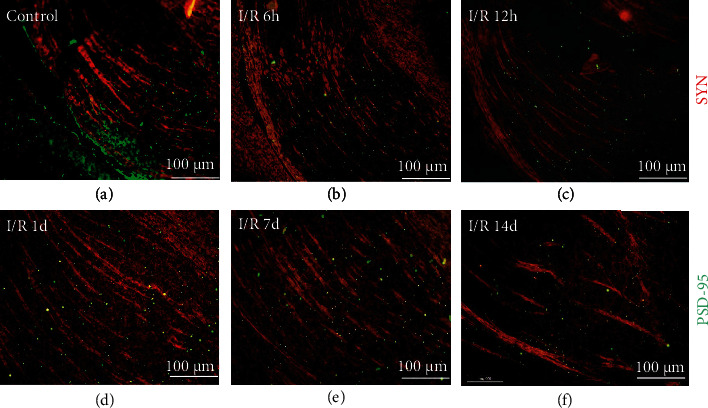
Immunofluorescence images of SYN and PSD-95 expression in dLGN after RI/R injury (100×, bar = 100 *μ*m). Representative photomicrographs taken at 100× immunofluorescence microscopy of rats' coronal plane of LGN on the left side, with (a) control group and (b) 6 hours; (c) 12 hours; (d) 1 day; 3 days; (e) 7 days; (f) 14 days post-RI/R injury. I/R-2 h: rats euthanized 2 hours post-RI/R injury; I/R-6 h: rats euthanized 6 hours post-RI/R injury; I/R-12 h: rats euthanized 12 hours post-RI/R injury; I/R-1 d: rats euthanized 1 day post-RI/R injury; I/R-1 d: rats euthanized 3 days post-RI/R injury; I/R-7 d: rats euthanized 7 days post-RI/R injury; I/R-14 d: rats euthanized 14 days post-RI/R injury.

*(2) SYN and PSD-95 Expression in dLGN*. The resulting Immunofluorescence images revealed that dLGN is spindle-shaped on the coronal section; SYN was fluorescently labeled in red and distributed in the axoplasm of nerve fibers. Retinal termini were stained and present red lines in Figure 9, according to the location of the RGC projections reported in the Claire Bc study [27]. PSD-95 was fluorescently labeled in green and distributed at each axon terminus of nerve fibers like stars. In I/R-6 h, I/R-12 h, and I/R-1d groups, the distribution of retinal termini had no significant changes when compared with control groups (Figure 9, control, I/R 6 h, I/R 12 h, and I/R 1 d). At 7 days to 14 days after injury, discontinuous changes occur in the nerve fibers of dLGN, and the number of nerve fibers was significantly smaller in the same field of view (Figure 9, I/R 7 d and I/R 14 d).

**Figure 10:**
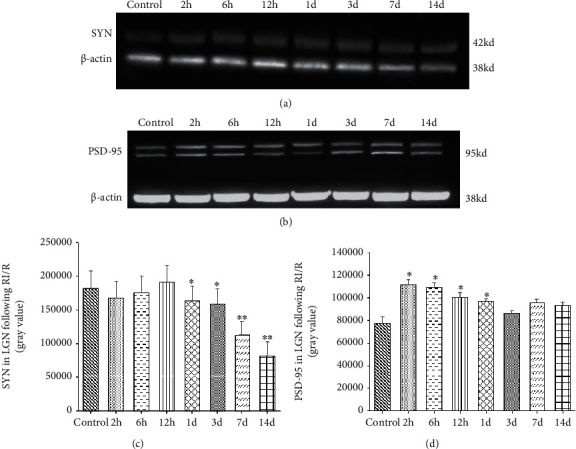
Western blot result of SYN and PSD-95 expression in LGN after RI/R injury. (a) 2 h: rats euthanized 2 hours post-RI/R injury; 6 h: rats euthanized 6 hours post-RI/R injury; 12 h: rats euthanized 12 hours post-RI/R injury; 1 d: rats euthanized 1 day post-RI/R injury; 3 d: rats euthanized 3 days post-RI/R injury; 7 d: rats euthanized 7 days. Synaptophysin (SYN) remains unchanged at 2 hours to 12 hours after RI/R compared to the control group. The SYN expression was significantly reduced in the retina at 1 day to 14 days after RI/R. (b) PSD-95 was significantly increased in the retina at 2 hours to 1 day after RI/R compared to the control group. From 3 days to 14 days, the PSD-95 expression showed a downward trend. (c) Use the Image J analysis software to determine the average gray value of the SYN expression in LGN. (d) Use the Image J analysis software to determine the average gray value of the PSD-95 expression in LGN. ^∗^*P* < 0.05 indicates that there were statistical differences when compared with the control groups. ^∗∗^*P* < 0.01 indicates that there were significant statistical differences when compared with the control groups.

Western blot (WB) analysis showed the expression of these two synaptic active proteins in the whole dLGN. SYN with the molecular weight of 38 kd remained unchanged from 2 hours to 12 hours after injury (*P* > 0.05). Then, from 1 day after the injury, the expression of SYN began to decrease (*P* < 0.05). In I/R-1 d, I/R-3 d, I/R-7 d, and I/R-14 d groups, SYN expression was significantly lower than the control group (*P* = 0.017, 0.003, 0.034, 0.001) (Figures 10(a) and 10(c)). And PSD-95 with a molecular weight of 95 kd was detected to be increased early after injury. From 2 hours to 1 day after the injury, the expression of PSD-95 was significantly higher than the control group (*P* = 0.031, 0.022, 0.017, 0.007). Then, from 1 d after injury, the expression of PSD-95 began to decrease (*P* < 0.05), and this downward trend continued from 1 d to 14 d (Figures 10(b) and 10(d)).

## 4. Discussion

Transneuronal degeneration has been proved to be associated with the pathological processes of a variety of neurological diseases, including Alzheimer's disease [6], amyotrophic lateral sclerosis [14], and brain trauma [8]. For the visual signal pathway, the significant loss of relay neurons in M and P layers of dLGN connected to the glaucomatous eye [29], which increased linearly with the loss of retinal afferent fibers, while Hendrickson et al. reported neuronal atrophy in dLGN after injury in the visual cortex [30]. These evidences suggest that primary neuron injury visual pathway presents anterograde and retrograde transneuronal degeneration on synaptic-linked distant neurons. It has been demonstrated that intervention measures that only compete against the mechanisms of in situ RGC death cannot satisfactorily recover the degenerated visual function following RI/R. Regrettably, previous research merely focused on preventing RGCs from death [27, 31], while ignoring the transneuronal degeneration following RI/R [32].

Presently, we found that relay cell edema, intracellular mitochondrial proliferation, and the increase in the number of presynaptic vesicles and mitochondria in IPL were emerged in the early stage after RI/R injury (2 hours to 12 hours, RGCs did not appear to be significantly lost). This shows that in the early stage of RI/R injury, abnormal mitochondria after an injury have appeared in the upper and lower neurons of RGCs. As the main place of cell productivity, mitochondria are prone to abnormal ultrastructural changes in the face of pathological factors such as ischemia and hypoxia [33]. The proliferation and hypertrophy of mitochondria are the typical manifestations of increased energy consumption after cell injury. Alexei put forward the lysosome mitochondrial axis-mediated cell death theory in 2010. This theory expounds that the change of mitochondrial membrane permeability can cause the change of lysosome permeability through ROS and/or Bcl-2 family-dependent manner and finally lead to cell death [34]. We found in the late stage after RI/R injury, mitochondrial dissolution in the presynaptic membrane of IPL and OPL also appeared, and then, bipolar and photoreceptor cells began to undergo apoptosis. Based on these experimental evidences, we speculated the early hyperplasia mitochondria may contribute to the apoptosis of bipolar and photoreceptor cells through the lysosomal-mitochondrial axis. This may be the reason for significant alterations in distal neurons after removal of RGC situ damage.

In addition, we observed that in the early stage of RI/R injury, synapses in IPL also showed significant plasticity changes. It is manifested by the formation of new small synapses, the increase in the number of presynaptic vesicles and the widening of the synaptic active band. This indicates that presynaptic structures and functions from the neurons in ONL are flourishing at this time. Previous studies have shown that the early newborn synapses are established rapidly after injury [35]. And by changing the expression level of synaptic active protein [22, 24], adjusting the level of cytokines [19–21], or by changing the content of mitochondria to transmit damage signals [23], the distal neurons are affected. These are the compensatory responses of neurons in early response to injury signals. Park et al. described in the chronic ocular hypertension model that RGCs showed more complex dendritic structure and more axon bundles than the control group 4 weeks after the induction of high intraocular pressure and formed more synaptic connections with bipolar cells [22]. These phenomena suggest that neurons in INL attempt to compensate for postinjury signal transmission by establishing more synaptic connections with RGCs. Interestingly, although the activity of presynaptic-related structures and functions in IPL in the early stage (2 h-12 h) after RI/R injury was observed in this study, there was no significant change in postsynaptic-related structures and functions. We speculate that as the earliest and most severely damaged kind of retinal neurons after RI/R injury (under the electron microscope, vacuolation and dissolution of organelles were observed in the RGCs' cytoplasm in the early stage after injury), the damaged/stressed RGCs could not respond to the increased presynaptic activity of bipolar cells. This asynchronous expression of this synaptic active protein may not only affect the effective signal transmission but also increase the energy consumption of upper neurons in RGCs. This may be one of the important reasons why patients with acute angle-closure glaucoma still have progressive loss of vision after the ocular hypertension injury is relieved. Of course, the mechanisms by which synaptic plasticity changes cause transneuronal degeneration remains to be further explored.

Previous studies reported that the relay cell soma in the LGN after RGC injury in the glaucoma model was atrophied, accompanied by the loss of nerve fibers. We also observed transneuronal changes in LGN in the early postinjury period, which were specifically manifested as neuronal edema, myelinated nerve demyelination, and new synapse formation. RI/R injury affects the transport function of ATP-dependent Na+/K+ pump on the cell membrane [36], which leads to intracellular sodium ion accumulation, cell edema, and volume increase. In this study, it was found that the early edema response was not only limited to neurons in the retina but also involved LGN neurons. Demyelination of myelinated nerves is a typical manifestation of neurodegenerative changes. The mechanism of demyelination involves oxidative stress theory [37], mitochondrial dysfunction theory, and excitotoxin theory [38]. The nerve impulse conduction of myelinated nerve fibers completes the jumping conduction through the adjacent *Nodes of Ranvier*. The thicker the myelin sheath and the longer the nodal body, the faster the conduction velocity. The change in demyelination makes the conduction rate of the nerve fiber significantly reduced [39]. Chang et al. [40] also detected an increase in the level of myelin alkaline protease in LGN after injury in the rat ocular hypertension model and believed that the degree of demyelination of LGN neurons was positively correlated with the degree of retinal damage.

In addition, similar alterations in synaptic active protein also appeared in the synapses between RGCs and relay cells in dLGN in the early stage after injury. Specifically, the expression of postsynaptic component PSD-95 in dLGN increased, while the expression of presynaptic vesicle protein SYN remained unchanged. Combined with previous studies, we believe that transneuronal degeneration in LGN after RI/R injury is still closely related to the changes in synaptic plasticity. Although the unsynchronous changes of synaptotagmins in LGN are similar to those in IPL, the changes in LGN are symmetrical with those in IPL. Relay cells showed a change process similar to bipolar cells, including the increase of synaptic vesicle protein, the widening of the synaptic active band, and the proliferation and hypertrophy of mitochondria. Evangelho et al. also mentioned that the anterograde axoplasmic transport function of injured RGCs was insufficient, and RGCs were in a state of dysfunction from soma to axon [41]. This may explain why PSD-95, an increased postsynaptic component of relay cells, lacks the response to the presynaptic components of RGCs.

In conclusion, our present study suggests that transneuronal degeneration exists in RI/R injury. Synaptic structures in the retina and LGN probably be associated with transneuronal degeneration through asynchronous changes of active proteins and mitochondrial abnormalities. This transneuronal degeneration may be one of the key reasons for the progressive deterioration of visual function after the injury is removed. By intervening in the early alterations of adjacent neurons and synapses to RGCs after RI/R injury, it will provide a new idea/inspiration for the recovery of visual function after ischemic retinopathy.

## Figures and Tables

**Table 1:** Mean density of RGCs using CTB retrograde tracer (*n* = 5).

Groups	Control	I/R-2 h	I/R-6 h	I/R-12 h	I/R-1 d	I/R-3 d	I/R-7 d	I/R-14 d
MD-RGCs (cells/mm^2^)	2325 ± 133	2450 ± 154	2350 ± 170	2457 ± 152	1500 ± 140	1475 ± 182	1425 ± 145	1137 ± 112
*F*		-626.52	-126.75	-661.24	4124.43	4499.64	5124.46	5939.16
*P*		0.825	1.581	1.304	<0.001^∗∗^	<0.001^∗∗^	<0.001^∗∗^	<0.001^∗∗^

Means ± standard error of the mean unless otherwise stated. MD-RGCs: mean density of labeled retinal ganglion cells. I/R-2 h: rats euthanized 2 hours post-RI/R injury; I/R-6 h: rats euthanized 6 hours post-RI/R injury; I/R-12 h: rats euthanized 12 hours post-RI/R injury; I/R-1 d: rats euthanized 1 day post-RI/R injury; I/R-1 d: rats euthanized 3 days post-RI/R injury; I/R-7 d: rats euthanized 7 days post-RI/R injury; I/R-14 d: rats euthanized 14 days post-RI/R injury. ^∗^*P* < 0.05 indicates that there were statistical differences when compared with the control groups. ^∗∗^*P* < 0.01 indicates that there were significant statistical differences when compared with the control groups.

**Table 2:** The thickness of retinal IPL and OPL in adult SD rats (*μ*m; *n* = 5).

	Control	2 h	6 h	12 h	1 d	3 d	7 d	14 d
IPL	54.03 ± 6.78	96.39 ± 8.30	119.79 ± 5.82	133.89 ± 6.65	75.66 ± 7.01	68.22 ± 6.98	58.83 ± 6.45	43.7 ± 7.11
*F*		-23.735	-38.086	-46.734	-11.021	-6.458	-0.699	2.447
*P*		<0.001^∗∗^	<0.001^∗∗^	<0.001^∗∗^	0.084	0.707	1.143	0.032^∗^
OPL	11.16 ± 0.91	15.06 ± 1.17	17.04 ± 1.32	16.35 ± 1.44	15.27 ± 1.93	12.39 ± 1.77	11.88 ± 1.21	11.34 ± 0.84
*F*		-31.631	-46.44	-46.730	-6.168	-6.977	-2.983	0.889
*P*		0.048∗	0.025^∗^	0.007^∗^	0.044^∗^	0.776	1.581	1.330

Means ± standard error of the mean unless otherwise stated. 2 h: rats euthanized 2 hours post-RI/R injury; 6 h: rats euthanized 6 hours post-RI/R injury; 12 h: rats euthanized 12 hours post-RI/R injury; 1 d: rats euthanized 1 day post-RI/R injury; 3 d: rats euthanized 3 days post-RI/R injury; 7 d: rats euthanized 7 days post-RI/R injury; 14 d: rats euthanized 14 days post-RI/R injury. ^∗^*P* < 0.05 indicates that there were statistical differences when compared with the control groups. ^∗∗^*P* < 0.01 indicates that there were significant statistical differences when compared with the control groups.

## Data Availability

The experimental data used to support the findings of this study are included within the article.

## Supplementary Materials

Supplementary Materials Supplementary Figure 1: stretched retinal image taken by immunofluorescence microscopy (40×). Sampling method for counting RGC numbers per fascicle. Each retina was taken at 5 images at high magnification (400x) visual field in every quadrant (superior, inferior, temporal, and nasal sides) along 1~1.5 mm from the ONH. The mean of 20 images represents the mean density of one stretched retina.
