# Supplementary figures and images for: Transneuronal Degeneration in the Visual Pathway of Rats following Acute Retinal Ischemia/Reperfusion
Source: Dis Markers. 2021 Dec 7;2021:2629150. doi: 10.1155/2021/2629150 (PMC8670974; doi:10.1155/2021/2629150)

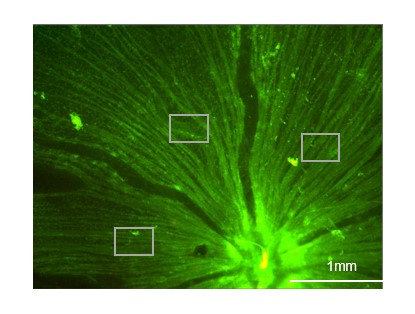

Supplement: Supplementary Materials — Supplementary Figure 1: stretched retinal image taken by immunofluorescence microscopy (40×). Sampling method for counting RGC numbers per fascicle. Each retina was taken at 5 images at high magnification (400x) visual field in every quadrant (superior, inferior, temporal, and nasal sides) along 1~1.5 mm from the ONH. The mean of 20 images represents the mean density of one stretched retina. [file 2629150.f1.jpg]
